# GDF11 Alleviates Pathological Myocardial Remodeling in Diabetic Cardiomyopathy Through SIRT1-Dependent Regulation of Oxidative Stress and Apoptosis

**DOI:** 10.3389/fcell.2021.686848

**Published:** 2021-06-28

**Authors:** Han-Zhao Zhu, Li-Yun Zhang, Meng-En Zhai, Lin Xia, Yu Cao, Lu Xu, Kai-Feng Li, Li-Qing Jiang, Heng Shi, Xiang Li, Ye-Nong Zhou, Wei Ding, Dong-Xu Wang, Er-He Gao, Jin-Cheng Liu, Shi-Qiang Yu, Wei-Xun Duan

**Affiliations:** ^1^Department of Cardiovascular Surgery, The First Affiliated Hospital, The Air Force Medical University, Xi’an, China; ^2^Department of Cardiovascular Surgery, General Hospital of Northern Theater Command, Shenyang, China; ^3^Department of Chinese Materia Medica and Natural Medicines, School of Pharmacy, The Air Force Medical University, Xi’an, China; ^4^Basic Medical Teaching Experiment Center, Basic Medical College, The Air Force Medical University, Xi’an, China; ^5^Center for Translational Medicine, Temple University School of Medicine, Philadelphia, PA, United States

**Keywords:** growth differentiation factor 11, diabetic cardiomyopathy, sirtuin 1, oxidative stress, apoptosis

## Abstract

Growth differentiation factor 11 (GDF11) is a member of the transforming growth factor β superfamily that alleviates cardiac hypertrophy, myocardial infarction, and vascular injury by regulating oxidative stress, inflammation, and cell survival. However, the roles and underlying mechanisms of GDF11 in diabetic cardiomyopathy (DCM) remain largely unknown. In this study, we sought to determine whether GDF11 could prevent DCM. After establishing a mouse model of diabetes by administering a high-fat diet and streptozotocin, intramyocardial injection of an adeno-associated virus was used to achieve myocardium-specific GDF11 overexpression. GDF11 remarkably improved cardiac dysfunction and interstitial fibrosis by reducing the levels of reactive oxygen species and protecting against cardiomyocyte loss. Mechanistically, decreased sirtuin 1 (SIRT1) expression and activity were observed in diabetic mice, which was significantly increased after GDF11 overexpression. To further explore how SIRT1 mediates the role of GDF11, the selective inhibitor EX527 was used to block SIRT1 signaling pathway, which abolished the protective effects of GDF11 against DCM. *In vitro* studies confirmed that GDF11 protected against H9c2 cell injury in high glucose and palmitate by attenuating oxidative injury and apoptosis, and these effects were eliminated by SIRT1 depletion. Our results demonstrate for the first time that GDF11 protects against DCM by regulating SIRT1 signaling pathway.

## Introduction

Diabetic cardiomyopathy (DCM) is one of the most serious complications of diabetes mellitus (DM), with high mortality and morbidity worldwide. It is characterized by pathological cardiac remodeling in the absence of coronary atherosclerosis, hypertension, or other cardiovascular diseases (Yancy et al., 2013; Wang and Hill, 2015). According to epidemiological data, it is estimated that the number of people with diabetes will rise to 300 million in 2025, and cardiac dysfunction occurs in nearly 50% of these patients (King et al., 1998; Boudina and Abel, 2007). Thus, DCM is an intractable problem. Over the past few decades, multiple mechanisms have been implicated in DCM progression, including myocardial fibrosis, inflammation, and cardiomyocyte loss, with elevated oxidative stress as a major contributor (Zhang et al., 2017; Riehle and Bauersachs, 2019). Hyperglycemia and hyperlipidemia act as trigger factors for DCM, and both contribute to a burst of reactive oxygen species (ROS) overproduction. Sustained increased levels of ROS disrupt the redox balance and react with proteins, nucleic acids, and lipids, inducing oxidative stress and ultimately resulting in myocardial apoptosis and interstitial fibrosis (Wilson et al., 2018). Therefore, the development of new therapeutic approaches to restrain ROS overproduction is urgent for patients with DCM.

Growth differentiation factor 11 (GDF11) is a member of the transforming growth factor β superfamily. It is broadly expressed in the body and present at particularly high levels in the kidneys, spleen, and heart, and it plays vital roles in a variety of physiological and pathological states (Schafer et al., 2016; Walker et al., 2016). Decreased serum GDF11 is associated with age-related cardiac hypertrophy, skeletal muscle regeneration, and neurogenic injury (Loffredo et al., 2013; Katsimpardi et al., 2014; Egerman et al., 2015). In addition, increasing the GDF11 level has cardioprotective effects in myocardial infarction and ischemic reperfusion injury, as well as in a myocardial pressure overload model (Du et al., 2017; Harper et al., 2018; Li et al., 2020). However, the underling mechanisms by which GDF11 regulates these pathological processes are unknown. Recent studies have focused on the crucial function of GDF11 in regulating ROS generation, as it also activates multiple antioxidant molecules to prevent oxidative damage (Wang et al., 2018; Su et al., 2019; Zhou et al., 2019). These findings underscore the potential of GDF11 in protecting against cardiac injury in DCM; however, this has not yet been investigated.

Sirtuin 1 (SIRT1) is a member of the sirtuin family. It has nicotinamide adenine dinucleotide (NAD^+^)-dependent deacetylase activity and regulates oxidative stress, inflammation, and cardiomyocyte survival during myocardial dysfunction (Bindu et al., 2016; Han et al., 2020). Cardiac SIRT1 expression is markedly decreased in diabetes, and our previous work showed that enhancing SIRT1 expression and deacetylase activity can restrict ROS release and upregulate downstream antioxidant proteins, such as superoxide dismutase (SOD), the transcription factor forkhead box O1, and heme oxygenase 1 (HO1) (Yu et al., 2014; Waldman et al., 2019; Ren et al., 2020). Intriguingly, all of these antioxidant molecules are also regulated by GDF11 (Lu et al., 2019; Su et al., 2019; Zhou et al., 2019). In addition, recent studies have demonstrated a tight correlation between GDF11 and SIRT1; however, whether GDF11 attenuates oxidative stress by modulating SIRT1 remains unclear (Opstad et al., 2019, 2020; Okada and Okada, 2020). Therefore, we aimed to explore the potential effects and underlying mechanisms of GDF11 on DCM.

## Materials and Methods

### Animals and Experimental Protocols

All animal procedures were performed in compliance with the Guide for the Care and Use of Laboratory Animals published by the United States National Institutes of Health (NIH publication no. 86-23, revised 1996) and with the approval of the Air Force Medical University Experimental Animal Research Committee. Male C57BL/6 mice (6–8 weeks old, 20–25 g) were obtained from the Experimental Animal Center of the Air Force Medical University and housed in cages at 22°C with a 12 h light/dark cycle.

The diabetic model was generated as previously described (Li K. et al., 2019). In brief, mice were fed a high-fat diet (HFD) for 1 month and then intraperitoneally injected with a low dose (60 mg/kg) of streptozotocin (STZ; Sigma-Aldrich, St. Louis, MO, United States) dissolved in citrate buffer (pH 4.5) for three consecutive days. After 2 weeks, mice with fasting blood glucose (FBG) levels >13.9 mmol/L (measured *via* the tail vein) were considered diabetic. Subsequently, intraperitoneal glucose tolerance tests (IPGTTs) and intraperitoneal insulin tolerance tests (IPITTs) were performed to estimate the tolerance of the diabetic mice to glucose and insulin. After successful establishment of diabetes, non-diabetic and diabetic mice were treated with adeno-associated viruses (AAV) containing either an empty vector or GDF11 cDNA (Hanbio Technology, Shanghai, China, 2 × 10^12^ viral genomes/mL) *via* intramyocardial injections into three points in the left ventricle, with a total injection volume of 24 μL. In addition, some mice were administered 20 mg/kg of the selective SIRT1 inhibitor EX527 (Sigma-Aldrich, MO, United States) diluted in 0.5% dimethyl sulfoxide twice daily by intraperitoneal injection. The mice were divided into eight groups (*n* = 15 each): (1) control (Con), (2) control with empty vector AAV treatment (Con + AAV-null), (3) control with AAV-GDF11 treatment (Con + AAV-GDF11), (4) DM, (5) DM with empty vector AAV treatment (DM + AAV-null), (6) DM with AAV-GDF11 treatment (DM + AAV-GDF11), (7) DM with EX527 treatment (DM + EX527), and (8) DM with AAV-GDF11 and EX527 treatment (DM + AAV-GDF11 + EX527). Mice were fed a normal diet or an HFD for 4 months and then killed. Heart tissues were collected for follow-up experiments (Supplementary Figure 1A).

### Cell Culture and Treatments

H9c2 cardiomyocytes obtained from Tiancheng Technology (Shanghai, China) were cultured in Dulbecco’s modified Eagle’s medium (HyClone, Logan, UT, United States) containing normal glucose (NG, 5.5 mmol/L) and supplemented with 10% fetal bovine serum and 1% penicillin/streptomycin at 37°C in a humidified atmosphere (95% air and 5% CO_2_). To mimic diabetes *in vitro*, H9c2 cardiomyocytes were cultured in high glucose (HG, 25 mmol/L glucose) with palmitate (Pal, 200 μmol/L) for 24 h to induce injury. H9c2 cells were transfected with adenoviruses encoding GDF11 (Ad-GDF11, 2.0 × 10^10^ plaque forming units [PFU]/mL) or an empty vector (Ad-null, 2.0 × 10^10^ PFU/mL; Hanbio Technology) for 6 h to overexpress GDF11 before HG and Pal treatment. To deplete SIRT1, H9c2 cells were transfected with SIRT1 small interfering (si)RNA (Hanbio, Nanjing, Jiangsu, China) for 24 h using Lipofectamine 3000 (Invitrogen, Carlsbad, CA, United States). The SIRT1 siRNA sequences were as follows: sense, 5′-CCA GUA GCA CUA AUU CCA ATT-3′ and antisense, 5′-UUG GAA UUA GUG CCA CUG GTT-3′, as described previously (Zhang B. et al., 2018).

### Echocardiography

Cardiac function was measured using a Visual Sonics Vevo 770 ultrasound system (Toronto, ON, Canada), as previously described (Zhai et al., 2017a). Mice were anesthetized by inhalation of 1.0% isoflurane in oxygen with stable heart rates of 400–500 beats/min, and M-mode echocardiography of the left ventricular short axis was obtained at the level of the papillary muscles using a 30 MHz linear transducer. The parameters of left ventricular function measured included the left ventricle internal dimension in systole (LVIDs) and diastole (LVIDd), left ventricular ejection fraction (LVEF), and left ventricular fractional shortening (LVFS), which were calculated by computerized algorithms.

### Histological Analysis, Immunohistochemistry, and Immunofluorescence

Myocardial tissues were preserved in 4% paraformaldehyde, embedded in paraffin, and sectioned to 5 μm. Then, the sections were stained with hematoxylin and eosin (H&E) or Masson’s trichrome stain to observe changes in myocardial morphology and assess the degree of collagen deposition, respectively. To observe GDF11 and SIRT1 expression in myocardium, cardiac sections were incubated overnight with primary antibodies against GDF11 (R&D Systems, Abingdon, United Kingdom: 1:50) and SIRT1 (Abcam, Cambridge, MA, United States; 1:50) at 4°C and then with anti-mouse and anti-rabbit secondary antibodies for 2 h at room temperature, respectively, as previously described (Zhai et al., 2017b). H9c2 cells were also incubated with a primary antibody against GDF11 and anti-mouse secondary antibody. To further examine the collagen fiber content, after antigen retrieval and sealing non-specific binding sites, sections were stained overnight with primary antibodies against Collagen I (Abcam; 1:50) and Collagen III (Abcam; 1:50) at 4°C, washed three times with phosphate-buffered saline, and then incubated with anti-rabbit secondary antibody for 2 h at room temperature. The level of fibrosis revealed by Masson’s trichrome staining was calculated using Image-Pro 6.0 (Media Cybernetics, Bethesda, MD, United States).

### Malondialdehyde Content and SOD and Glutathione Peroxidase Activity Assays

The Malondialdehyde (MDA) content and SOD and Glutathione Peroxidase (GSH-Px) activities in the heart tissues were detected using enzyme-linked immunosorbent assay kits purchased from Nanjing Jiancheng Bioengineering Institute (Nanjing, Jiangsu, China), following the manufacturer’s instructions. A SpectraMax M5 Multi-Mode Microplate Reader (Molecular Devices, San Jose, CA, United States) was used to measure the signals by spectrophotometry.

### Determination of SIRT1 Activity

According to the manufacturer’s instructions, SIRT1 deacetylase activity was measured by a fluorometric assay kit (Enzo Life Sciences). The fluorescence of each sample was detected via using a SpectraMax M5 instrument with a 360 nm excitation wavelength and a 460nm emission wavelength.

### ROS Detection

The oxidative fluorescent dyes dihydroethidium (Invitrogen) and 2′,7′-dichlorofluorescein diacetate (DCFH-DA; Beyotime Institute of Biotechnology, Shanghai, China) were used to detect ROS levels in frozen myocardial sections and H9c2 cells, according to the manufacturers’ instructions, as previously described.^28^ Sections and H9c2 cells were observed under an Olympus FV1000 laser confocal microscope (Olympus, Tokyo, Japan). To quantify ROS generation, fluorescence intensity was measured using Image-Pro 6.0.

### Terminal Deoxynucleotidyl Transferase dUTP Nick End Labeling Assays

Cardiomyocyte apoptotic ratio was measured by transferase dUTP nick end labeling (TUNEL) staining using the *In Situ* Cell Death Detection Kit (Roche Molecular Biochemicals, Mannheim, Germany). Briefly, myocardial sections and H9c2 cells were TUNEL stained according to the manufacturer’s instructions and then counterstained with 4′,6′-diamidino-2-phenylindole. TUNEL-positive cells emitted green fluorescence. The apoptotic ratio was the number of TUNEL-positive cells to the total number of cardiomyocytes, calculated using Image-Pro 6.0.

### Western Blot Analysis

Lysate homogenates of whole tissues and cells were prepared as previously reported (Zhang B. et al., 2018). After total protein quantification with bicinchoninic acid, 10% sodium dodecyl sulfate-polyacrylamide gel electrophoresis was used to resolve the proteins, which were then transferred onto polyvinylidene fluoride membranes. The membranes were blocked with Tris-buffered saline containing Tween 20 (TBST; 50 mM Tris, 150 mM NaCl, 0.1% Tween 20, pH 7.6) and 5% skim milk for 2 h at room temperature. Then, the membranes were incubated overnight at 4°C with primary antibodies against GDF11 (1:1000), SIRT1 (Abcam; 1:1000), HO1 (Abcam; 1:1000), nuclear factor-erythroid 2-related factor 2 (Nrf2; Abcam; 1:1000), SOD2 (Cell Signaling Technology, Boston, MA, United States; 1:1000), Bax (Cell Signaling Technology; 1:1000), Bcl-2 (Cell Signaling Technology; 1:1000), Cleaved Caspase-3 (Cell Signaling Technology; 1:1000), NF-κB p65 (Cell Signaling Technology; 1:1000), Phospho-NF-κB p65 (p-p65; Cell Signaling Technology; 1:1000), gp91^*phox*^ (Proteintech, Rosemont, IL, United States; 1:1000), and GAPDH (Proteintech; 1:5000). After washing in TBST, the membranes were reacted with appropriate horseradish peroxidase-conjugated secondary antibodies (Proteintech; 1:5000) for 2 h. Finally, bands were visualized by enhanced chemiluminescence (Millipore, MA, United States), and Image Lab software (Bio-Rad Laboratories, Irvine, CA, United States) was used to measure the density of the immunoreactive bands.

### Statistical Analysis

Data are presented as the mean ± standard error of the mean. Differences between two groups were analyzed by one-way analysis of variance and *post hoc* Tukey tests using GraphPad Prism 8.0 (GraphPad Software Inc., San Diego, CA, United States). *P* < 0.05 was considered statistically significant.

## Results

### Establishment of the Diabetic Model

To ensure successful induction of the diabetic model, FBG and body weight (BW) measurements were obtained, and IPGTTs and IPITTs were performed after STZ injection. Diabetic mice had higher FBG levels and BW than non-diabetic mice (Supplementary Figures 1B,C). In addition, diabetic mice were sensitive to glucose and also tolerant to insulin (Supplementary Figures 1D,E).

### Overexpression of GDF11 in Myocardial Tissues and H9c2 Cells

Three months after intramyocardial injection of AAV-GDF11, the transfection efficiency was investigated by immunofluorescence and western blot. AAV-GDF11 treatment markedly elevated GDF11 expression in myocardial tissues, with a nearly 2.5-fold increase in GDF11 protein compared with that in the Con and DM groups (Supplementary Figures 2A–C). Notably, cardiac GDF11 expression was significantly lower in the DM group than in the Con group (Supplementary Figures 2B,C). Similar results were observed in H9c2 cells after Ad-GDF11 transfection (Supplementary Figures 2D–F).

### Overexpression of GDF11 Ameliorates Diabetes-Induced Cardiac Dysfunction

**FIGURE 1:**
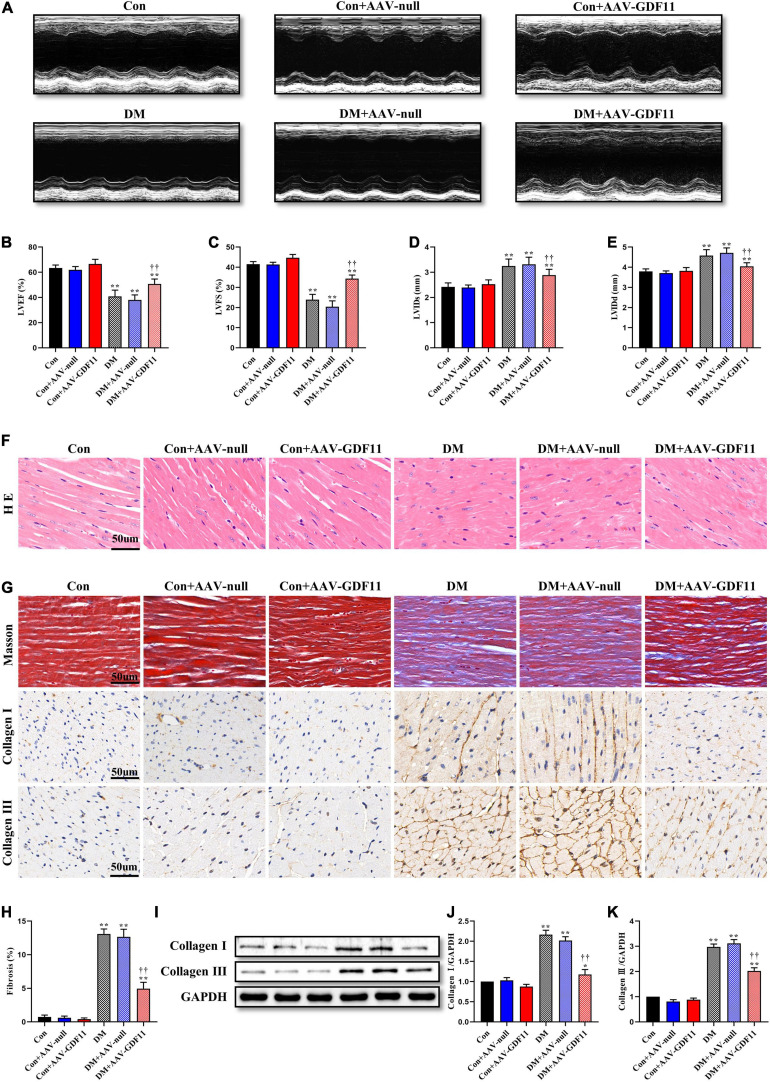
Myocardial GDF11 overexpression improved cardiac systolic function and attenuates interstitial fibrosis. **(A)** Representative images of M-mode echocardiography. **(B)** Left ventricular ejection fraction (LVEF). **(C)** Left ventricular fractional shortening (LVFS). **(D)** Left ventricle internal dimension in systole (LVIDs). **(E)** Left ventricle internal dimension in diastole (LVIDd). **(F)** Representative images of myocardial sections stained with hematoxylin and eosin (H&E) (scale bar = 50 μm). **(G)** Representative images of Masson’s trichrome staining and immunohistochemistry for Collagen I and Collagen III (scale bar = 50 μm). **(H)** Interstitial fibrosis in myocardial sections. **(I)** Representative blots of Collagen I and Collagen III. **(J)** Quantitative expression of Collagen I. **(K)** Quantitative expression of Collagen III. Data are presented as the mean ± SEM, *n* = 5 or 6 per group. *,**P < 0.05, 0.01 versus the Con group, ^††^*P* < 0.01 versus the DM group.

Diabetes-induced cardiac systolic dysfunction was evident by the end of our study, with declines in echocardiography-derived indicators of left ventricular systolic function (LVEF and LVFS). Upregulation of GDF11 markedly augmented the above parameters in diabetic mice; however, AAV-null diabetic mice developed severe heart failure (Figures 1A–C). Additionally, compared with mice in the Con group, those in the DM and DM-AAV groups exhibited remarkable decreases in LVIDs and LVIDd, which were also increased by myocardial GDF11 overexpression (Figures 1D,E). Cardiac function did not differ between the Con + AAV-GDF11 and Con groups. Taken together, these results indicate a function for GDF11 in the prevention of cardiac dysfunction.

### Overexpression of GDF11 Prevents Adverse Myocardial Remodeling in DCM

To further investigate the effects of GDF11 on myocardial remodeling, H&E and Masson’s trichrome staining were used to detect pathological structure changes. Diabetic mice had disordered myocardial structures and abnormal cardiomyocyte morphologies, and these phenotypes were alleviated in the DM + AAV-GDF11 group (Figure 1F). In addition, in the hearts of diabetic mice, excessive fibrosis was observed together with higher levels of Collagen I and Collagen III than in the hearts of mice in the Con group (Figures 1G–K). Delivery of AAV-GDF11 to the myocardium not only dramatically attenuated collagen deposition but also effectively downregulated Collagen I and Collagen III expression, revealing the potential of GDF11 in ameliorating cardiac fibrosis (Figures 1G–K). AAV-null delivery had no effect on myocardial remodeling in non-diabetic or diabetic mice, and AAV-GDF11 delivery had no effect in non-diabetic mice.

### GDF11-Induced Attenuations in Myocardial Oxidative Stress and Apoptosis Are Mediated by SIRT1 Upregulation

**FIGURE 2:**
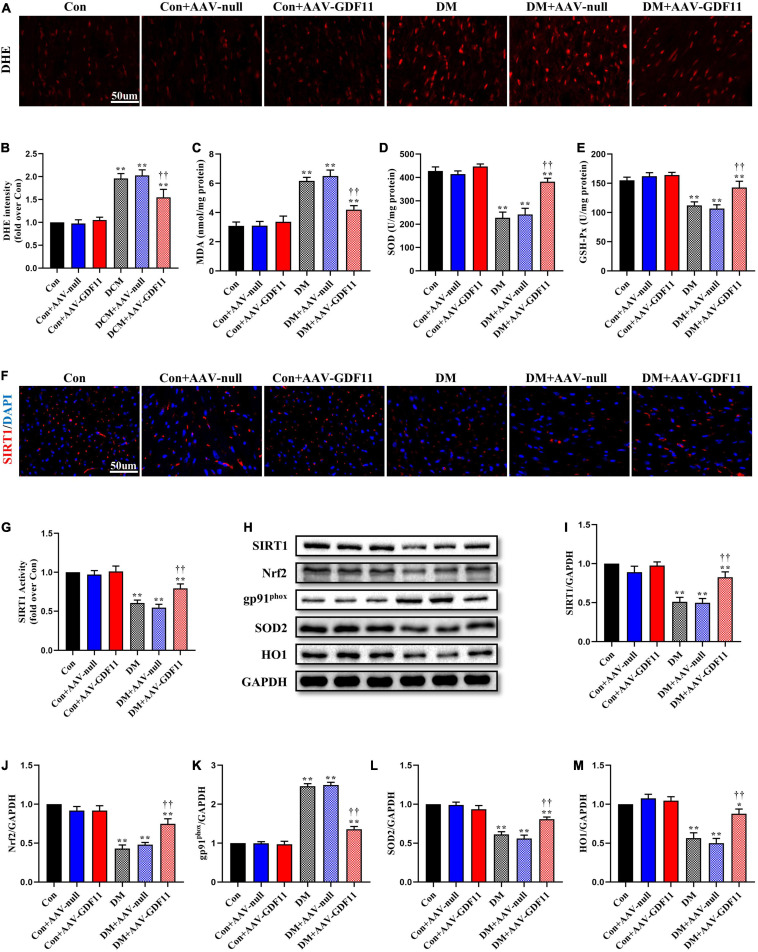
GDF11 inhibited oxidative damage in diabetic cardiomyopathy (DCM) by upregulating SIRT1 expression. **(A)** Representative images of dihydroethidium (DHE) staining (scale bar = 50 μm). **(B)** DHE fluorescence intensities. **(C)** Malondialdehyde (MDA) content. **(D)** Superoxide dismutase (SOD) activity. **(E)** Glutathione peroxidase (GSH-Px) activity. **(F)** Representative images of SIRT1 immunofluorescence in heart tissue. **(G)** The deacetylase activity of SIRT1. **(H)** Representative blots of SIRT1, Nrf2, gp91^*phox*^, SOD2, and HO1. **(I)** Quantitative expression of SIRT1. **(J)** Quantitative expression of Nrf2. **(K)** Quantitative expression of gp91^*phox*^. **(L)** Quantitative expression of SOD2. **(M)** Quantitative expression of HO1. Data are presented as the mean ± SEM, *n* = 5 or 6 per group. *,**P < 0.05, 0.01 versus the Con group, ^††^*P* < 0.01 versus the DM group.

Redox imbalance is a landmark of diabetes-induced myocardial injury. ROS generation was markedly higher in the myocardium of the DM group than the Con group, while GDF11 overexpression significantly attenuated the ROS level (Figures 2A,B). The activities of two pivotal antioxidant enzymes, SOD and GSH-Px, were visibly decreased in the hearts of DM mice (Figures 2C–E), which also had increased levels of MDA, a lipid peroxidation product, indicating oxidative stress damage. AAV-GDF11 administration largely reversed these changes. To determine the effects of GDF11 on the expression of oxidative stress related proteins, the level and activity of SIRT1 and the expressions of its downstream effectors Nrf2, SOD2, gp91^*phox*^, and HO1 were analyzed. AAV-GDF11-treated diabetic mice displayed remarkably higher level and activity of SIRT1 than the DM group, along with increases in Nrf2, SOD2, and HO1. Furthermore, GDF11 inhibited the diabetes-induced upregulation of the pro-oxidant gp91^*phox*^ (Figures 2F–M).

**FIGURE 3:**
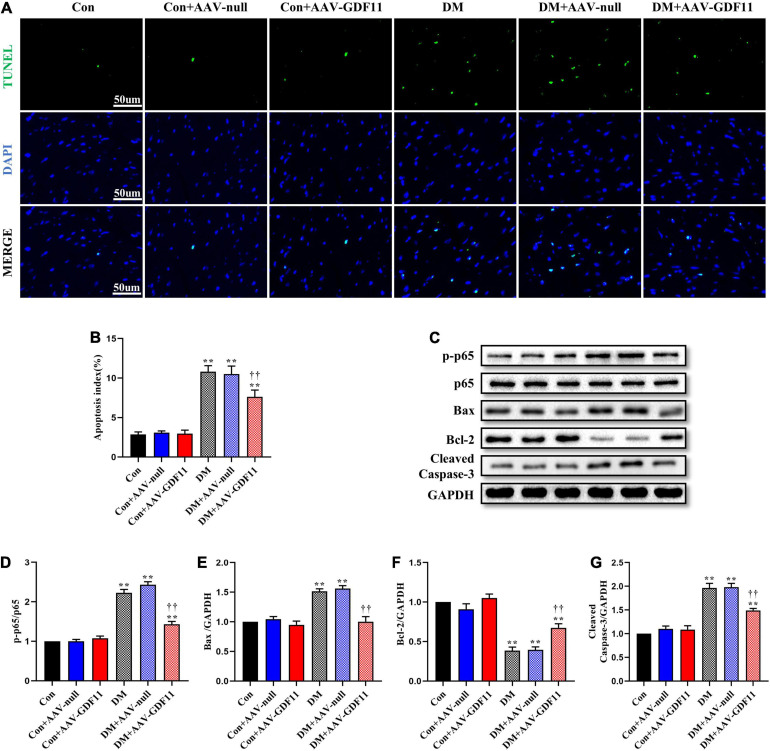
GDF11 prevented diabetes-induced cardiomyocyte apoptosis. **(A)** Representative images of TUNEL staining (scale bar = 50 μm). **(B)** Apoptotic ratio. **(C)** Representative blots of p-p65, p65, Bax, Bcl-2, and Cleaved Caspase-3. **(D)** Quantitative analysis of the ratio of p-p65 to p65. **(E)** Quantitative expression of Bax. **(F)** Quantitative expression of Bcl-2. **(G)** Quantitative expression of Cleaved Caspase-3. Data are presented as the mean ± SEM, *n* = 5 per group. **P < 0.01 versus the Con group, ^††^*P* < 0.01 versus the DM group.

TUNEL staining showed increased cardiomyocyte apoptosis in diabetic mice compared with that in control mice (Figures 3A,B) as well as significant increases in p-p65, Bax and Cleaved Caspase-3 and a decrease in Bcl-2, an antiapoptotic protein (Figures 3C–G). Mice in the DM + AAV-GDF11 group had lower cardiomyocyte apoptotic ratio than mice in the DM group (Figures 3A,B). Consistently, they also displayed increased Bcl-2 and decreased Bax, Cleaved Caspase-3 and p65 phosphorylation (Figures 3C–G). Notably, these differences were not observed between the Con and Con + AAV-null groups or between the DM and DM + AAV-null groups. These results illustrate that GDF11 may exert its protection against oxidative stress and cardiomyocyte apoptosis by regulating SIRT1 expression.

### SIRT1 Signaling Pathway Mediates the Cardioprotective Effects of GDF11 Against DCM

**FIGURE 4:**
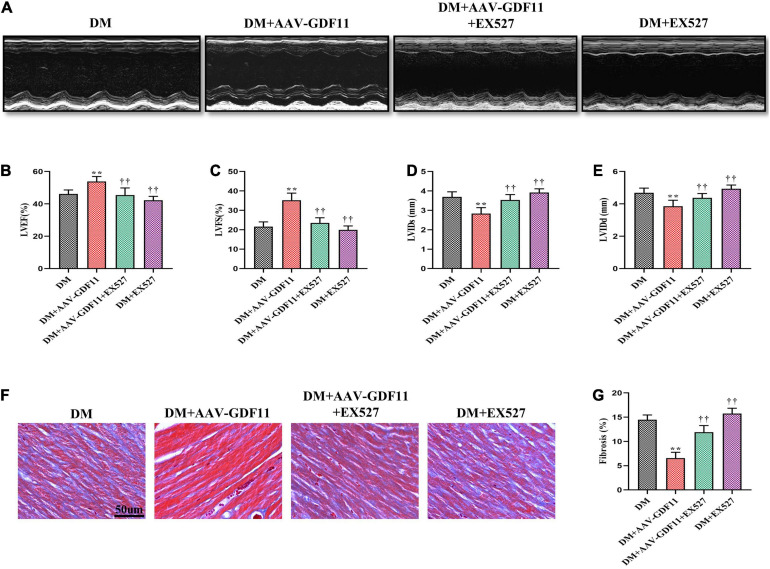
Treatment with EX527 eliminated the positive effects of GDF11 on myocardial function and cardiac fibrosis. **(A)** Representative images of M-mode echocardiography. **(B)** Left ventricular ejection fraction (LVEF). **(C)** Left ventricular fractional shortening (LVFS). **(D)** Left ventricle internal dimension in systole (LVIDs). **(E)** Left ventricle internal dimension in diastole (LVIDd). **(F)** Representative images of Masson’s trichrome staining (scale bar = 50 μm). **(G)** Interstitial fibrosis in myocardial sections. Data are presented as the mean ± SEM, *n* = 6 per group. **P < 0.01 versus the DM group, ^††^*P* < 0.01 versus the DM + AAV-GDF11 group.

Subsequently, to verify whether GDF11 improves cardiac function *via* the SIRT1 signaling pathway, we inhibited SIRT1 using EX527. GDF11-induced increases in LVEF and LVFS observed in diabetic mice were diminished after treatment with EX527, as were the changes in the LVIDs and LVIDd (Figures 4A–E). Similarly, attenuation of cardiac fibrosis by GDF11 was also offset by inhibiting SIRT1 expression (Figures 4F–G). However, treatment of diabetic mice with EX527 alone had little effect on these phenotypes.

### SIRT1 Signaling Pathway Propagates the Beneficial Effects of GDF11 by Inhibiting Oxidative Stress and Cardiomyocyte Apoptosis in DCM

**FIGURE 5:**
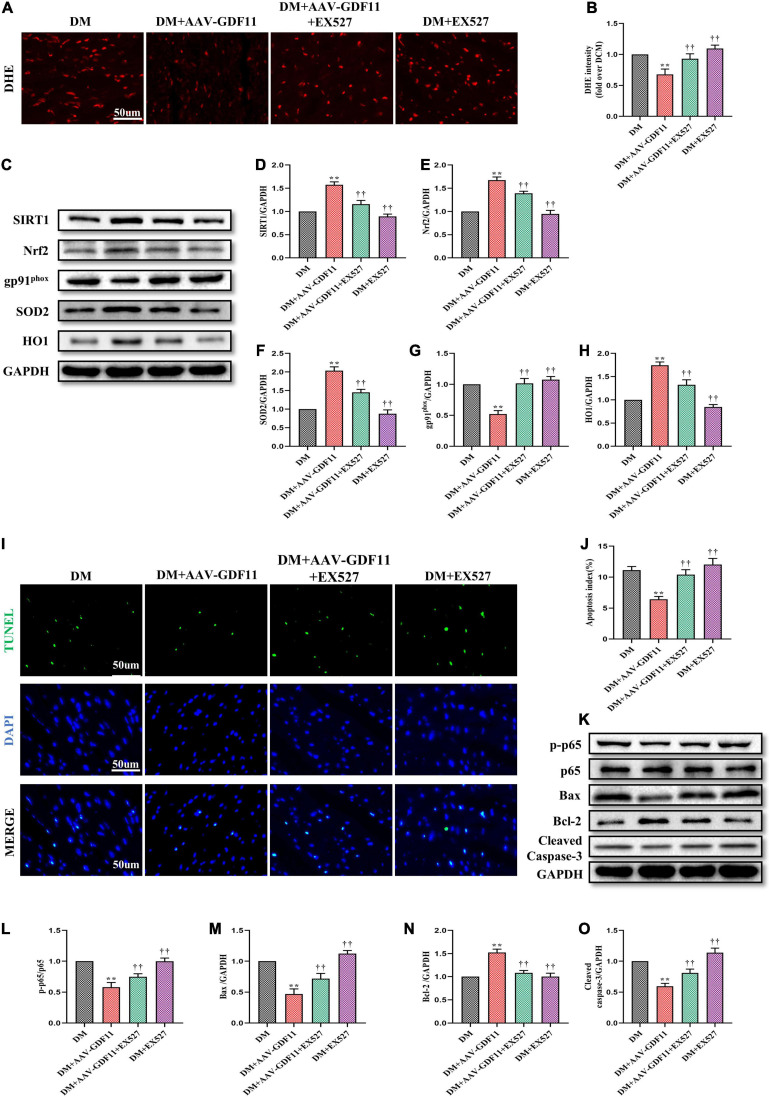
SIRT1 inhibition abolished the antioxidative and antiapoptotic functions of GDF11 in diabetic cardiomyopathy (DCM). **(A)** Representative images of dihydroethidium (DHE) staining (scale bar = 50 μm). **(B)** DHE fluorescence intensities. **(C)** Representative blots of SIRT1, Nrf2, gp91^*phox*^, SOD2, and HO1. **(D)** Quantitative expression of SIRT1. **(E)** Quantitative expression of Nrf2. **(F)** Quantitative expression of gp91^*phox*^. **(G)** Quantitative expression of SOD2. **(H)** Quantitative expression of HO1. **(I)** Representative images of TUNEL staining (scale bar = 50 μm). **(J)** Apoptotic ratio. **(K)** Representative blots of p-p65. p65, Bax, Bcl-2, and Cleaved Caspase-3. **(L)** Quantitative analysis of the ratio of p-p65 to p65. **(M)** Quantitative expression of Bax. **(N)** Quantitative expression of Bcl-2. **(O)** Quantitative expression of Cleaved Caspase-3. Data are presented as the mean ± SEM, *n* = 5 or 6 per group. **P < 0.01 versus the DM group, ^††^*P* < 0.01 versus the DM + AAV-GDF11 group.

Our *in vivo* data indicated a strong antioxidative relationship between GDF11 and SIRT1; therefore, we further explored whether GDF11 exerts its functions through the SIRT1 signaling pathway. Inhibiting SIRT1 expression largely reversed the reduction of ROS caused by GDF11 overexpression in diabetic mice (Figures 5A,B). EX527 not only inhibited an increase in the expression of SIRT1 after treating AAV-GDF11, but also reduced the activity of SIRT1 (Supplementary Figures 3A,B and Figures 5C,D). The increases in Nrf2, SOD2, and HO1 protein levels and the decrease in gp91^*phox*^ expression in diabetic mice treated with GDF11 were also eliminated by EX527 (Figures 5C,E–H). The SIRT1 inhibitor also blunted the decrease in cardiomyocyte apoptosis after GDF11 overexpression, as well as the changes in apoptotic proteins induced by AAV-GDF11 in diabetes (Figures 5I–O).

### Upregulation of GDF11 Activates SIRT1 to Protect Against HG- and Pal-Induced H9c2 Cell Injury

**FIGURE 6:**
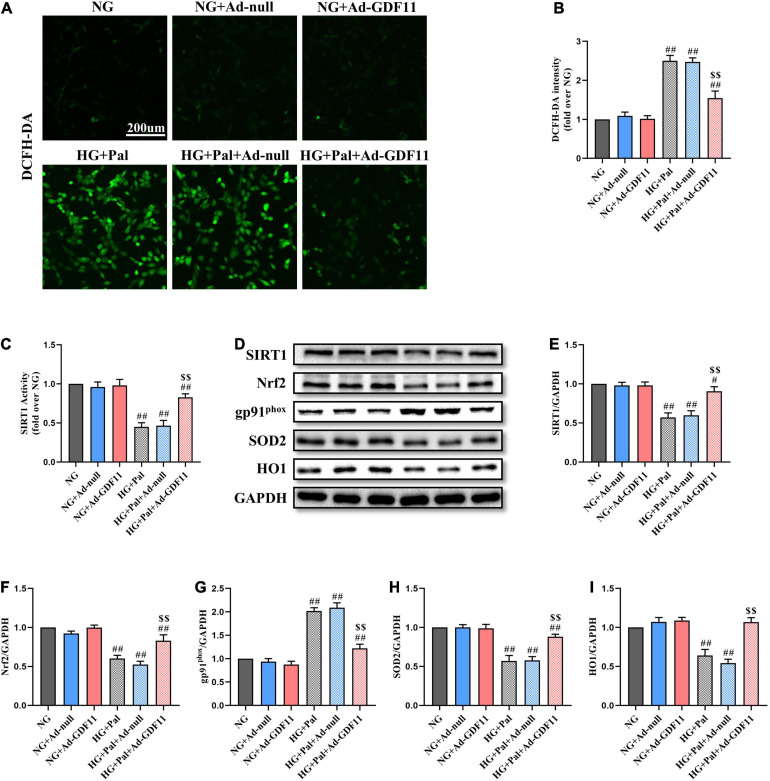
GDF11 overexpression attenuated high glucose (HG) and palmitate (Pal) induced reactive oxygen species (ROS) overproduction in H9c2 cells. **(A)** Representative images of 2’,7’-dichlorofluorescein diacetate (DCFH-DA) staining (scale bar = 200 μm). **(B)** DCFH-DA fluorescence intensities. **(C)** The deacetylase activity of SIRT1. **(D)** Representative blots of SIRT1, Nrf2, gp91^*phox*^, SOD2, and HO1. **(E)** Quantitative expression of SIRT1. **(F)** Quantitative expression of Nrf2. **(G)** Quantitative expression of gp91^*phox*^. **(H)** Quantitative expression of SOD2. **(I)** Quantitative expression of HO1. Data are presented as the mean ± SEM, *n* = 5 or 6 per group. ^#/##^*P* < 0.05/0.01 versus the NG group, ^$$^*P* < 0.01 versus the HG + Pal group.

Given the positive effects of GDF11 *in vivo*, we further explored whether GDF11 could activate SIRT1 to alleviate oxidative stress and cell death *in vitro*. After transfection with either Ad-null or Ad-GDF11, H9c2 cells were stimulated with HG and Pal for 24 h, at which point they exhibited increased ROS generation compared with that in the NG group. Intriguingly, the level of ROS was lower in the HG + Pal + Ad-GDF11 group than in the HG + Pal group, while Ad-GDF11 had no significant effect on the NG group (Figures 6A,B). In accordance with the results of *in vivo* experiments, Ad-GDF11 reversed the reductions in SIRT1 expression and activity, as well as antioxidative proteins caused by HG and Pal treatment, while also suppressing the gp91^*phox*^ level (Figures 6C–I).

**FIGURE 7:**
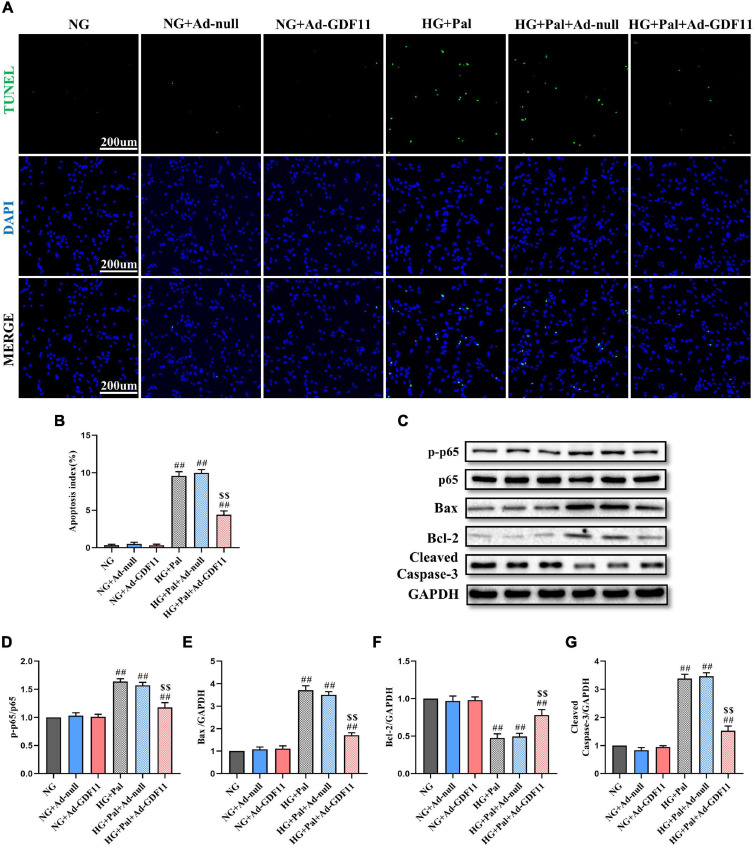
GDF11 decreased high glucose (HG) and palmitate (Pal) induced apoptosis *in vitro*. **(A)** Representative images of TUNEL staining (scale bar = 200 μm). **(B)** Apoptotic ratio. **(C)** Representative blots of p-p65, p65, Bax, Bcl-2, and Cleaved Caspase-3. **(D)** Quantitative analysis of the ratio of p-p65 to p65. **(E)** Quantitative expression of Bax. **(F)** Quantitative expression of Bcl-2. **(G)** Quantitative expression of Cleaved Caspase-3. Data are presented as the mean ± SEM, *n* = 5 or 6 per group. ^##^*P* < 0.01 versus the NG group, ^$$^*P* < 0.01 versus the HG + Pal group.

In addition, the loss of H9c2 cells after HG and Pal treatment was assessed by TUNEL staining and western blotting for relevant apoptotic proteins. GDF11 overexpression markedly decreased TUNEL-positive cells and downregulated p-p65, Bax, and Cleaved Caspase-3 in the HG + Pal + Ad-GDF11 group compared with that in the HG + Pal group, and increased Bcl-2 (Figures 7A–G). Notably, there were no differences between the NG and NG + Ad-null groups or between the HG + Pal and HG + Pal + Ad-null groups.

### Blocking SIRT1 Signaling Pathway Eliminates the Protective Effects of GDF11 in HG- and Pal-Treated H9c2 Cells

**FIGURE 8:**
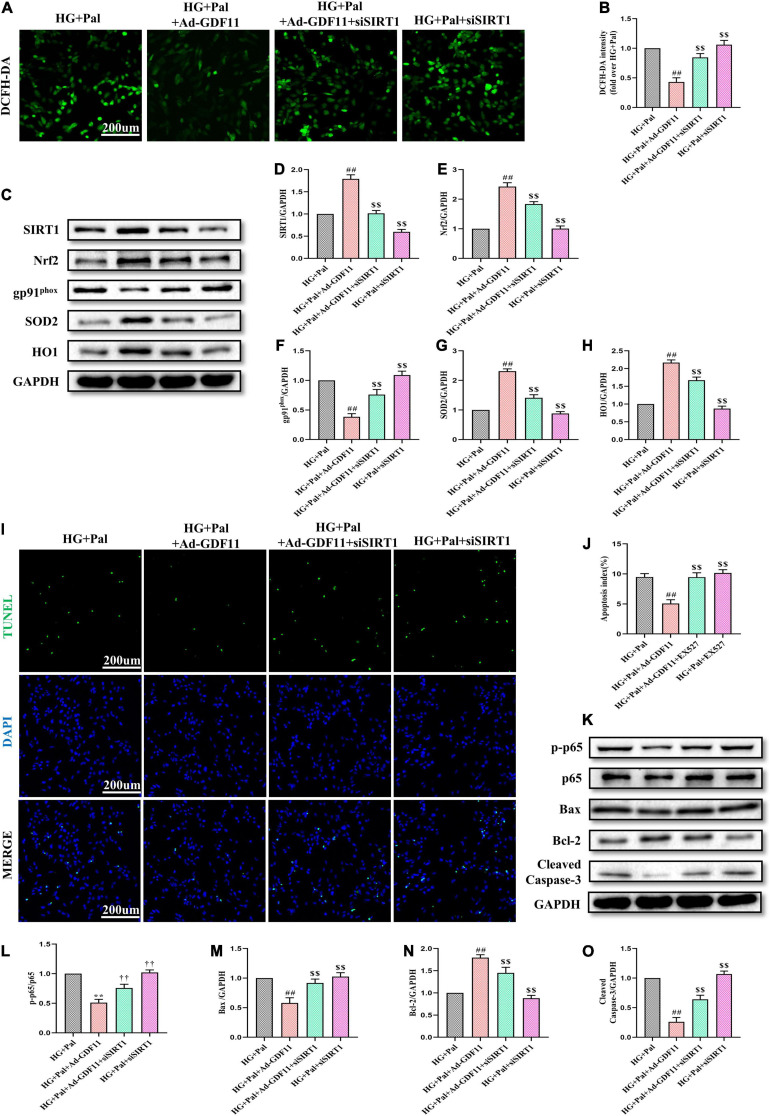
SIRT1 siRNA diminished GDF11-induced inhibition of oxidative stress and apoptosis in H9c2 cells. **(A)** Representative images of 2’,7’-dichlorofluorescein diacetate (DCFH-DA) staining (scale bar = 200 μm). **(B)** DCFH-DA fluorescence intensities. **(C)** Representative blots of SIRT1, Nrf2, gp91^*phox*^, SOD2, and HO1. **(D)** Quantitative expression of SIRT1. **(E)** Quantitative expression of Nrf2. **(F)** Quantitative expression of gp91^*phox*^. **(G)** Quantitative expression of SOD2. **(H)** Quantitative expression of HO1. **(I)** Representative images of TUNEL staining (scale bar = 200 μm). **(J)** Apoptotic ratio. **(K)** Representative blots of p-p65, p65, Bax, Bcl-2, and Cleaved Caspase-3. **(L)** Quantitative analysis of the ratio of p-p65 to p65. **(M)** Quantitative expression of Bax. **(N)** Quantitative expression of Bcl-2. **(O)** Quantitative expression of Cleaved Caspase-3. Data are presented as the mean ± SEM, *n* = 5 or 6 per group. ^##^*P* < 0.01 versus the HG + Pal group, ^$$^*P* < 0.01 versus the HG + Pal + Ad-GDF11 group.

To confirm the role of the SIRT1 signaling pathway in GDF11-mediated attenuations in oxidative stress and apoptosis in H9c2 cells, we depleted SIRT1 with siRNA (Supplementary Figures 4A–C). SIRT1 siRNA significantly abolished the effects of GDF11 on ROS levels and SIRT1, Nrf2, SOD2, HO1, and gp91^*phox*^ expression (Figures 8A–H). Consistent with the *in vivo* results, SIRT1 siRNA also elevated the number of TUNEL-positive cells in the HG + Pal + Ad-GDF11 group compared with that in the HG + Pal group, along with remarkable increases in the levels of p-p65, Bax and Cleaved Caspase-3 and a decrease in Bcl-2 (Figures 8I–O). However, SIRT1 siRNA had little effect on Ad-null H9c2 cells stimulated with HG and Pal.

## Discussion

Cardiovascular diseases are the leading cause of death in patients with diabetes, and specific treatments for DCM remain limited (Saeedi et al., 2019). Accumulating evidence has verified the protective effects of GDF11 against diabetes-related diseases (Li et al., 2017; Zhang J. et al., 2018). However, our study is the first to demonstrate that heart-specific overexpression of GDF11 confers cardioprotection against DCM, as confirmed by its antioxidative stress and antiapoptotic effects. GDF11 mediated these effects by regulating SIRT1 expression and may be a promising target for DCM treatment.

Cardiac dysfunction in DCM is usually attributed to pathological structures in the myocardium, which is characterized by extracellular collagen deposition and matrix remodeling (Asbun and Villarreal, 2006). Excessive collagen fiber formation and abnormal cardiomyocyte alignment increase the stiffness of the myocardium, affecting systolic and diastolic functions (Li et al., 2018). Consistent with our previous results, we observed collagen overproduction in STZ-induced diabetic mice compared with that in mice in the Con group, along with severe heart failure (Li K. et al., 2019). Notably, the GDF11 protein level was also significantly decreased in DCM, which may explain these adverse changes. GDF11 supplementation has been reported to diminish myocardial fibrosis in disease states. Harper et al. (2018) found that GDF11 contributes to a dose-dependent reduction in interstitial fibrosis under pressure overload and attenuates pathological cardiac hypertrophy. In addition, GDF11 can blunt the development of heart failure by reducing cardiac fibrosis after myocardial ischemia reperfusion injury and inhibits liver fibrosis by regulating hepatic progenitor cells (Du et al., 2017; Dai et al., 2020). In accordance with these results, our study revealed that upregulation of GDF11 markedly alleviated myocardial systolic function by elevating LVEF and LVFS and reduced fibrosis in the cardiac interstitium, including decreased expression of Collagen I and III. These data suggest that GDF11 plays a decisive role in reversing diabetes-induced cardiac remodeling by regulating the production of collagen fibers.

Oxidative stress, triggered by ROS overproduction, is a major characteristic of DCM (Ritchie and Abel, 2020). Because of dysregulation of glycolipid metabolism, the accumulation of lipid peroxidation products and advanced glycation end-products disrupts the balance between ROS formation and scavenging, ultimately inducing intracellular oxidative stress injury. Therefore, inhibiting ROS generation is crucial to treat DCM (Doenst et al., 2013). In our study, the ROS level was obviously increased in the hearts of diabetic mice compared with that in mice in the Con group. In addition, the levels of the antioxidative enzymes SOD and GSH-Px decreased after ROS stimulation, while those of MDA, a lipid peroxidation product indicative of failure of the antioxidative system, was increased, consistent with the results of previous investigations (Feng et al., 2019; Li C. et al., 2019). The effects of GDF11 on reversing age-related cardiac hypertrophy were initially discovered by parabiosis, and its identification as a rejuvenation factor led to further experiments revealing its antioxidative stress function (Loffredo et al., 2013). Onodera et al. (2017) demonstrated that GDF11 suppressed cigarette smoke extract-induced ROS activity in human fetal lung fibroblasts. Moreover, intraperitoneal injection of recombinant GDF11 into aged male mice not only enhanced the activities of antioxidative enzymes, including catalase, GSH-Px, and SOD, but also blocked the increase in MDA content (Zhou et al., 2019). We found that overexpression of GDF11 could attenuate excessive ROS generation *in vivo* and *in vitro* and recover the function of antioxidative systems, highlighting its potency in alleviating oxidative damage.

The number of cardiomyocytes present in cardiac tissue dictates the quality of cardiac contractility, and their loss due to oxidative stress invariably results in cardiac remodeling and dysfunction (Jia et al., 2018). Numerous studies have clarified the protective functions of GDF11 on cell survival, including attenuating apoptosis and pyroptosis. Mei et al. (2016) reported that GDF11 supplementation attenuated endothelial cell apoptosis during Pal treatment. Furthermore, overexpression of GDF11 in the serum could inhibit cardiomyocyte pyroptosis in acute myocardial infarction by targeting the transcription factor homeobox A3 (Li et al., 2020). Consistent with these results, our study revealed that GDF11 treatment markedly reduced the increase in TUNEL-positive cardiomyocytes in DCM and in H9c2 cells treated with HG and Pal. The antiapoptotic capacity of GDF11 induced strong upregulation of Bcl-2, together with decreases in Bax and Cleaved Caspase-3 via regulating the phosphorylation of p65. This is consistent with the results of a previous study demonstrating that GDF11 prevented mesenchymal stem cell apoptosis under hypoxic culture conditions by regulating these molecules (Zhao et al., 2020). Taken together, these observations support a role for GDF11 in preventing cell death in DCM.

Although the roles of GDF11 in mitigating oxidative stress and apoptosis have been thoroughly demonstrated, little is known regarding its downstream effectors. SIRT1, the founding member of the sirtuin family, is regarded as a major modulator of DCM, because of its antioxidative and antiapoptotic effects (Ma et al., 2017). Combined with previous evidence, the results in our study indicate that GDF11 and SIRT1 could dramatically inhibit oxidative damage by targeting the same antioxidative molecules, thereby reducing myocardial ROS (Liao et al., 2019; Su et al., 2019; Zhou et al., 2019). Moreover, quercetin treatment simultaneously upregulates GDF11 and SIRT1 expression in fibroblasts (Okada and Okada, 2020). In patients with coronary artery disease, whole blood GDF11 and SIRT1 expression levels were strongly correlated (Opstad et al., 2019). Hence, a vital question raised in our study is whether the role of GDF11 is to activate SIRT1. As expected, SIRT1 expression and activity were suppressed both in the hearts of the diabetic mice and in HG + Pal-treated H9c2 cells, while overexpression of GDF11 significantly activated SIRT1 and elevated its downstream molecules Nrf2, SOD2, and HO1. Similarly, GDF15, another member of the GDF family, can upregulate SIRT1 expression in lipopolysaccharide-induced acute lung injury (Song et al., 2020). We blocked the SIRT1 signaling pathway *in vivo* and *in vitro* using EX527 and SIRT1 siRNA, respectively. The suppression of SIRT1 signaling dramatically eliminated the protective effects of GDF11 against cardiac dysfunction and fibrosis. In addition, SIRT1 inhibition blunted the reduced ROS level in the myocardium and the upregulation of antioxidative molecules induced by GDF11 overexpression. Notably, cardiomyocyte apoptosis in DCM is also regulated by SIRT1 (Ding et al., 2015). We showed that the inhibitory effect of GDF11 against cardiomyocyte apoptosis was offset by blocking SIRT1 expression and activity both *in vivo* and *in vitro*. Thus, the results indicate that the beneficial roles of GDF11 in DCM are largely dependent on the activity of SIRT1 signaling pathway.

Taken together, our study reveals that GDF11 overexpression protects against diabetes-induced myocardial injury by attenuating cardiac fibrosis, oxidative stress, and cardiomyocyte apoptosis by activating the SIRT1 signaling pathway. These results provide a novel target for DCM treatment.

## Conclusion

Here, we confirmed a novel molecular target for DCM by which GDF11 attenuated cardiac dysfunction and reversed adverse myocardial remodeling in diabetes. The protective effects of GDF11 on DCM derived from the inhibition of oxidative stress and cardiomyocyte apoptosis via the SIRT1 signaling pathway. Overexpression of GDF11 provided a new strategy for diabetic patients to treat heart injury induced by hyperglycemia. Thus, GDF11 has the potential for clinical application in the future, but the further studies should focus on the function of GDF11 in clinical outcome.

## Data Availability Statement

The original contributions presented in the study are included in the article/Supplementary Material, further inquiries can be directed to the corresponding author/s.

## Ethics Statement

The animal study was reviewed and approved by the Air Force Medical University Experimental Animal Research Committee.

## Author Contributions

W-XD and S-QY designed the study. H-ZZ, L-YZ, M-EZ, LXi, WD, and YC performed the animal experiments. L-YZ, L-QJ, Y-NZ, and XL analyzed all the data. H-ZZ, LXu, W-XD, K-FL, and D-XW performed the cell experiments. H-ZZ, L-YZ, and M-EZ wrote the manuscript. S-QY, J-CL, and W-XD revised the manuscript. All authors read and approved the final manuscript.

## Conflict of Interest

The authors declare that the research was conducted in the absence of any commercial or financial relationships that could be construed as a potential conflict of interest.

## Supplementary Material

Supplementary Figure S1 Successful establishment of the diabetic model. **(A)** Experimental flow graph. **(B)** Body weight. **(C)** Fasting blood glucose (FBG) after 2-week STZ injection. **(D)** Intraperitoneal glucose tolerance tests (IPGTTs). **(E)** Intraperitoneal insulin tolerance tests (IPITTs). Data are presented as the mean ± SEM, *n* = 5 per group. ***P* < 0.01 versus the non-diabetic group.

Supplementary Figure S2 GDF11 overexpression was induced by AAV-GDF11 and Ad-GDF11 in myocardial tissues and H9c2 cells, respectively. **(A)** Representative images of GDF11 immunofluorescence in heart tissue. **(B)** Representative blot of GDF11. **(C)** Quantitative expression of GDF11. **(D)** Representative images of GDF11 immunofluorescence in H9c2 cells. **(E)** Representative blot of GDF11. **(F)** Quantitative expression of GDF11. Data are presented as the mean ± SEM, *n* = 5 per group. ***P* < 0.01 versus the Con group, ^††^*P* < 0.01 versus the DM group.

Supplementary Figure S3 EX527 blunted the increases in the expression and activity of SIRT1 in myocardium after GDF11 overexpression. **(A)** Representative images of SIRT1 immunofluorescence in heart tissue. **(B)** The deacetylase activity of SIRT1. Data are presented as the mean ± SEM, *n* = 5 or 6 per group. ***P* < 0.01 versus the DM group, ^††^*P* < 0.01 versus the DM + AAV-GDF11 group.

Supplementary Figure S4 The expression and activity of SIRT1 were inhibited by SIRT1 siRNA in H9c2 cells. **(A)** Representative images of SIRT1 immunofluorescence in H9c2 cells. **(B)** Representative blot of SIRT1. **(C)** The deacetylase activity of SIRT1 in H9c2 cells. Data are presented as the mean ± SEM, *n* = 5 or 6 per group. ***P* < 0.01 versus the Scramble siRNA group.
