# Supplementary figures and images for: GDF11 Alleviates Pathological Myocardial Remodeling in Diabetic Cardiomyopathy Through SIRT1-Dependent Regulation of Oxidative Stress and Apoptosis
Source: Front Cell Dev Biol. 2021 Jun 28;9:686848. doi: 10.3389/fcell.2021.686848 (PMC8273395; doi:10.3389/fcell.2021.686848)

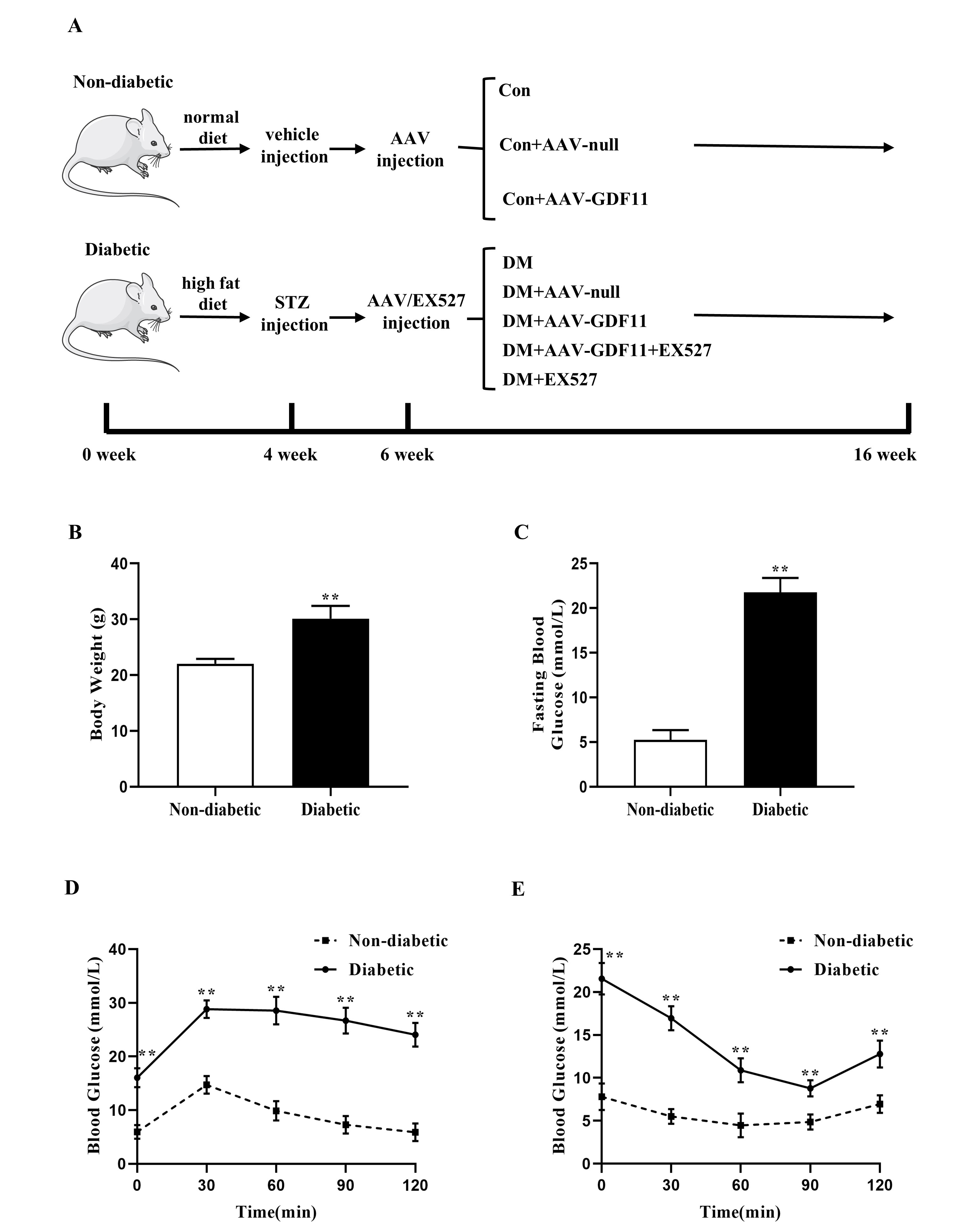

Supplement: Supplementary Figure S1 — Successful establishment of the diabetic model. (A) Experimental flow graph. (B) Body weight. (C) Fasting blood glucose (FBG) after 2-week STZ injection. (D) Intraperitoneal glucose tolerance tests (IPGTTs). (E) Intraperitoneal insulin tolerance tests (IPITTs). Data are presented as the mean ± SEM, n = 5 per group. **P < 0.01 versus the non-diabetic group. [file Image_1.JPEG]

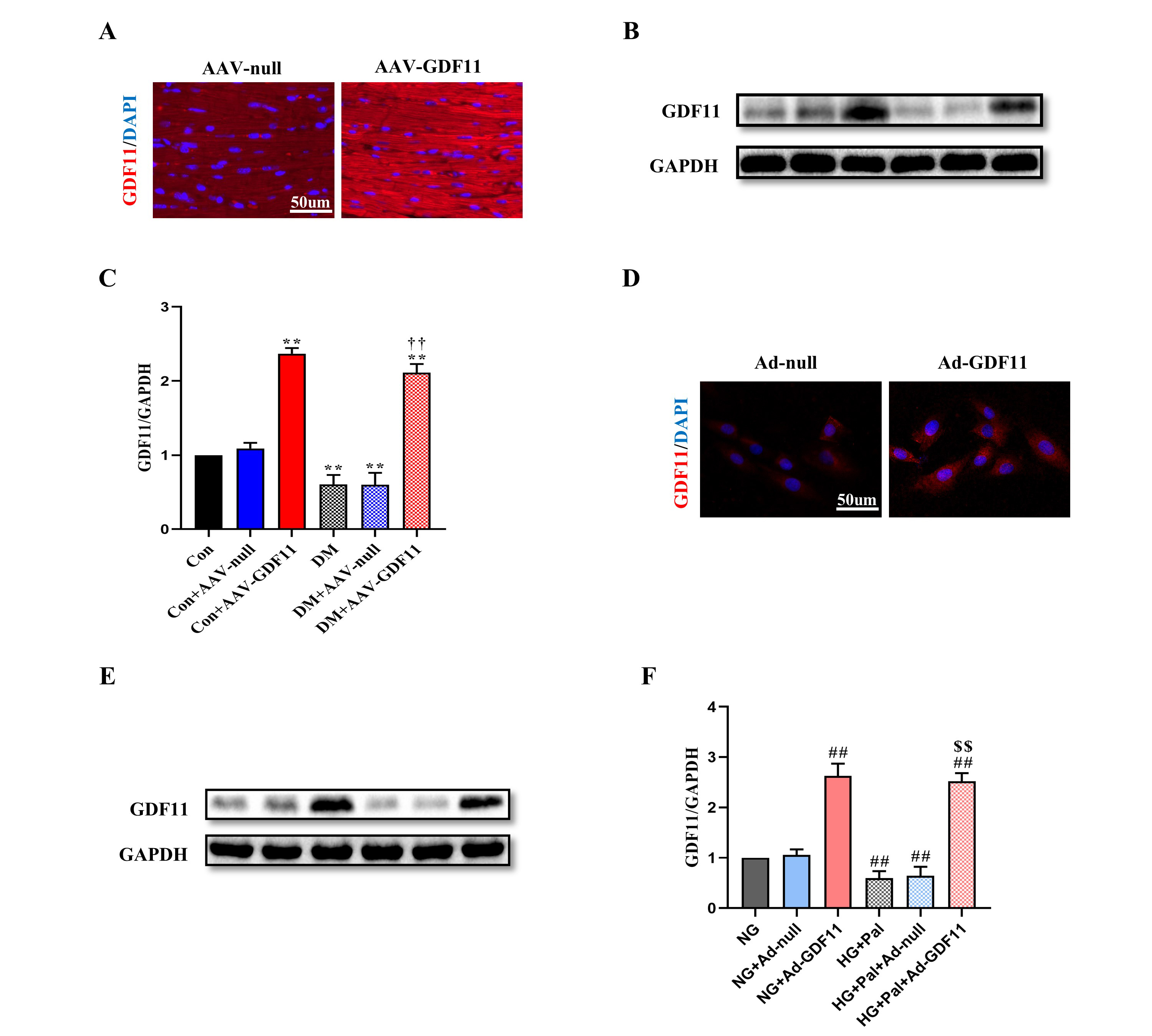

Supplement: Supplementary Figure S2 — GDF11 overexpression was induced by AAV-GDF11 and Ad-GDF11 in myocardial tissues and H9c2 cells, respectively. (A) Representative images of GDF11 immunofluorescence in heart tissue. (B) Representative blot of GDF11. (C) Quantitative expression of GDF11. (D) Representative images of GDF11 immunofluorescence in H9c2 cells. (E) Representative blot of GDF11. (F) Quantitative expression of GDF11. Data are presented as the mean ± SEM, n = 5 per group. **P < 0.01 versus the Con group, ††P < 0.01 versus the DM group. [file Image_2.JPEG]

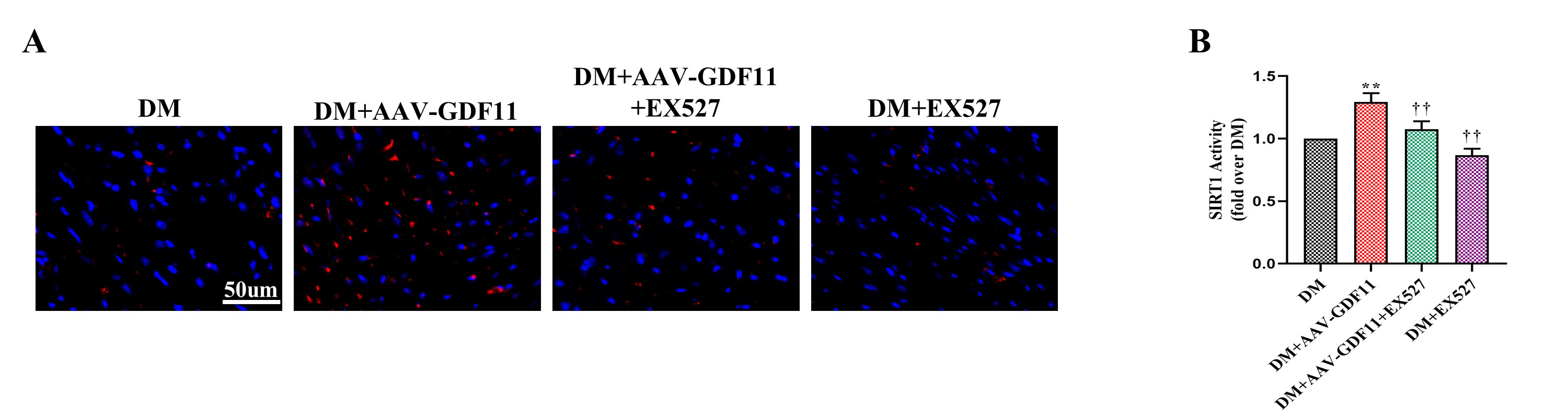

Supplement: Supplementary Figure S3 — EX527 blunted the increases in the expression and activity of SIRT1 in myocardium after GDF11 overexpression. (A) Representative images of SIRT1 immunofluorescence in heart tissue. (B) The deacetylase activity of SIRT1. Data are presented as the mean ± SEM, n = 5 or 6 per group. **P < 0.01 versus the DM group, ††P < 0.01 versus the DM + AAV-GDF11 group. [file Image_3.JPEG]

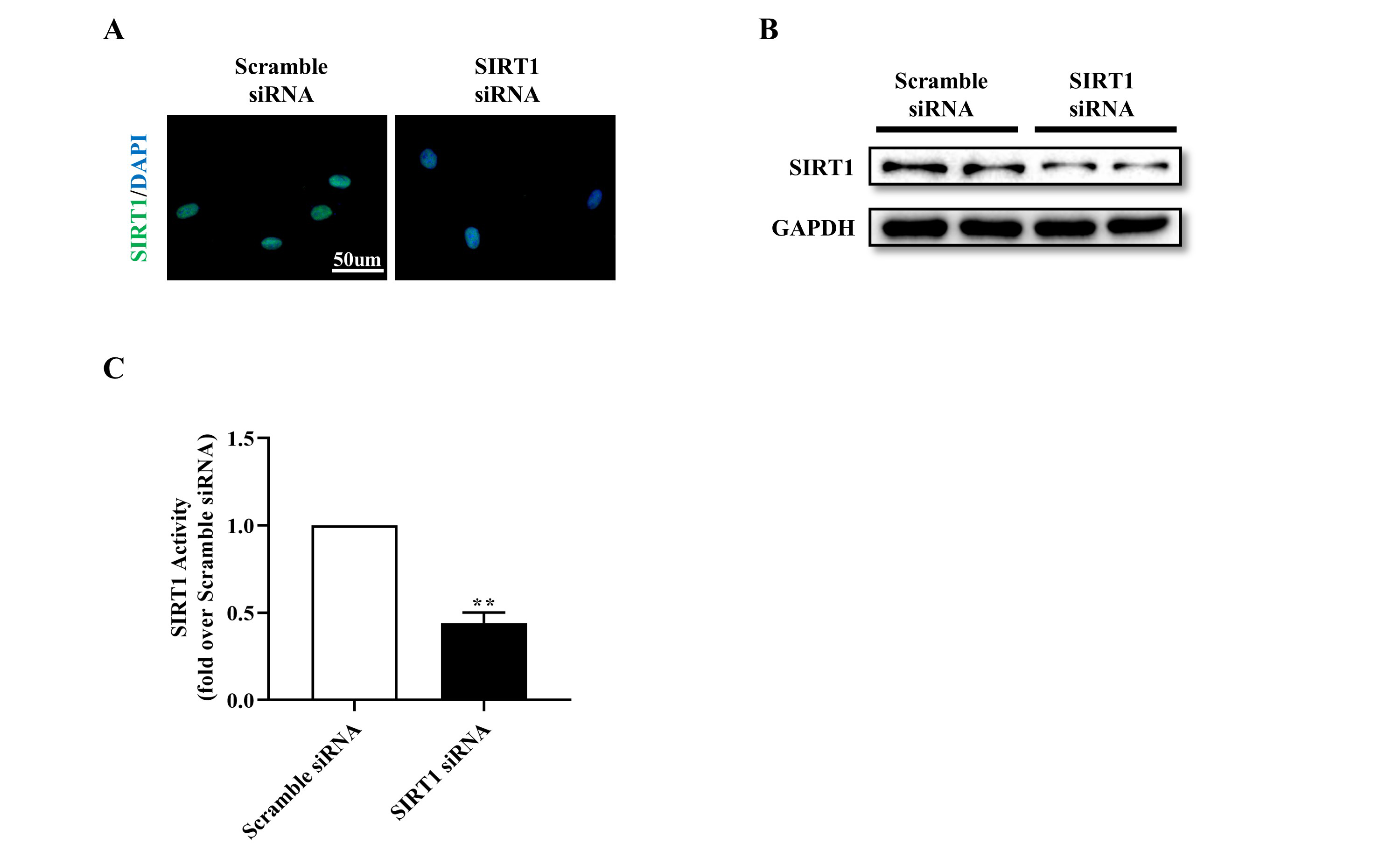

Supplement: Supplementary Figure S4 — The expression and activity of SIRT1 were inhibited by SIRT1 siRNA in H9c2 cells. (A) Representative images of SIRT1 immunofluorescence in H9c2 cells. (B) Representative blot of SIRT1. (C) The deacetylase activity of SIRT1 in H9c2 cells. Data are presented as the mean ± SEM, n = 5 or 6 per group. **P < 0.01 versus the Scramble siRNA group. [file Image_4.JPEG]
